# Decline in small mammal species richness in coastal‐central California, 1997–2013

**DOI:** 10.1002/ece3.10611

**Published:** 2023-12-10

**Authors:** Yadav P. Ghimirey, William D. Tietje, Anne Y. Polyakov, James E. Hines, Madan K. Oli

**Affiliations:** ^1^ Department of Wildlife Ecology and Conservation University of Florida Gainesville Florida USA; ^2^ Department of Environmental Science, Policy, and Management University of California Berkeley California USA; ^3^ United States Geological Survey Eastern Ecological Science Center Laurel Maryland USA

**Keywords:** climate variability, climate‐driven species richness, colonization‐extinction dynamics, imperfect detection, multiseason occupancy modeling

## Abstract

The richness and composition of a small mammal community inhabiting semiarid California oak woodland may be changing in response to climate change, but we know little about the causes or consequence of these changes. We applied a capture‐mark‐recapture model to 17 years (1997–2013) of live trapping data to estimate species‐specific abundances. The big‐eared woodrat was the most frequently captured species in the area, contributing 58% of total captures. All small mammal populations exhibited seasonal fluctuations, whereas those of the California mouse, brush mouse, and pinyon mouse declined during the study period. We also applied a multispecies dynamic occupancy model to our small mammal detection history data to estimate species richness, occupancy (ψ), detection (*p*), local extinction (ϵ), and colonization (γ) probabilities, and to discern factors affecting these parameters. We found that ψ decreased from 0.369 ± 0.088 in 1997 to 0.248 ± 0.054 in 2013; γ was lower during the dry season (May–September) than the wet season (October–April) and was positively influenced by total seasonal rainfall (slope parameter, β = 0.859 ± 0.371; 95% CI = 0.132–1.587). Mean mammalian species richness decreased from 11.943 ± 0.461 in 1997 to 7.185 ± 0.425 in 2013. With highly variable climatic patterns expected in the future, especially increased frequency and intensity of droughts, it is important to monitor small mammal communities inhabiting threatened California oak woodlands.

## Graphical abstract

We used dynamic occupancy models to understand the temporal variation of community parameters such as species richness, extinction, and colonization dynamics.

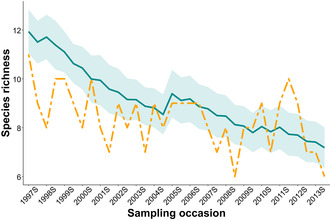

## 1 INTRODUCTION

Species richness, the number of species present at a given place and time, is an important indicator of biodiversity and an Essential Biodiversity Variable (Chaudhary et al., 2022; Gotelli & Colwell, 2010; Hillebrand et al., 2018). However, the traditional approach to estimating species richness as the number of species detected at a given place and time underestimates species richness because it fails to account for imperfect detection of species present on the study area (Guillera‐Arroita et al., 2019; MacKenzie et al., 2002). Failure to account for imperfect detection can cause bias in the species richness estimates, potentially leading to erroneous conclusions (Boulinier et al., 2001; Nichols et al., 2006). However, recent advances in occupancy models that utilize detection–nondetection data offer a framework for incorporating imperfect detection while estimating species richness (Dorazio et al., 2006; MacKenzie et al., 2002, 2017). In particular, multiseason or dynamic occupancy (DO) models have emerged as powerful tools for estimating species and community‐level parameters (e.g., colonization and local extinction probabilities) and modeling factors and processes influencing these parameters (MacKenzie et al., 2003, 2017). Importantly, these models permit estimation of species richness and allow an assessment of factors affecting species richness while also accounting for imperfect detection (Dorazio et al., 2006; Kéry & Royle, 2020; MacKenzie et al., 2017).

Even when species richness remains constant, community composition and relative abundances of constituent species may change over time. For example, a small mammal community of the Kluane Lake region in the Yukon, Canada, was composed of 10 species, with four species dominating the biomass (Krebs et al., 2019). During the 1970s, northern red‐backed vole (*Myodes rutilus*) and eastern deer mouse (*Peromyscus maniculatus*) were equally abundant. However, between the 1970s and 2000s, the relative abundance of red‐backed voles increased by 22%, with a corresponding decrease in the relative abundance of deer mouse by 22%. More recently, red‐backed voles have been the most dominant species, representing 63% of all small mammal captures. Thus, a major shift in the relative abundances of constituent species occurred without a detectable change in species richness (Krebs et al., 2019). Similar changes in community composition have been reported for an Afro‐montane forest bird community in Rwanda (Morton et al., 2022), and for a bird community in the Great Lake's region in Minnesota (Parody et al., 2001).

Although the population ecology of some small mammals inhabiting the coastal California oak woodlands is well understood (e.g., Polyakov et al., 2021; Srivathsa et al., 2019; Tietje et al., 2018), we know little about many aspects of the changes in community composition over time, and the biotic and abiotic factors influencing these changes. As such, estimates of small mammal species richness that account for imperfect detection are currently not available for oak woodlands of coastal California. Here, we use 17 years (1997–2013) of biannual capture‐mark‐recapture (CMR) data to determine the composition and species richness of a small mammal community inhabiting a semiarid oak woodland in coastal‐central California. We used the proportion of captures of each species as a measure of their relative abundance in the community (e.g., Krebs et al., 2019). For species with adequate sample sizes, we applied CMR analyses (Williams et al., 2002) to estimate abundance (time‐specific population size). Finally, we applied the DO model to our detection–nondetection data to estimate small mammal species richness and to evaluate factors and processes affecting species richness, including species occupancy (ψ), local extinction (ϵ), and colonization (γ) probabilities.

We know that the ecology of many small mammal species inhabiting semiarid habitats is strongly seasonal and rainfall driven (Kelt et al., 1996; Meserve et al., 2011); thus, we expected small mammal community parameters to also vary seasonally, and be affected by rainfall patterns. Hence, we hypothesized that (i) ϵ would be lower, and γ and species richness higher during the wet season (October–April) than the dry season (May–September); and (ii) ϵ would be negatively affected, while γ and species richness would be positively affected by current and past seasonal rainfall and by the El Niño index (Oceanic Niño Index, ONI). With the predictions that severe droughts are likely to be more frequent and more intense in coastal California (Garfin et al., 2013; Moser et al., 2012), our findings will inform community‐level biodiversity monitoring in coastal California, and aid in the formulation or implementation of small mammal conservation measures.

## 2 MATERIALS AND METHODS

### 2.1 Study area

**FIGURE 1:**
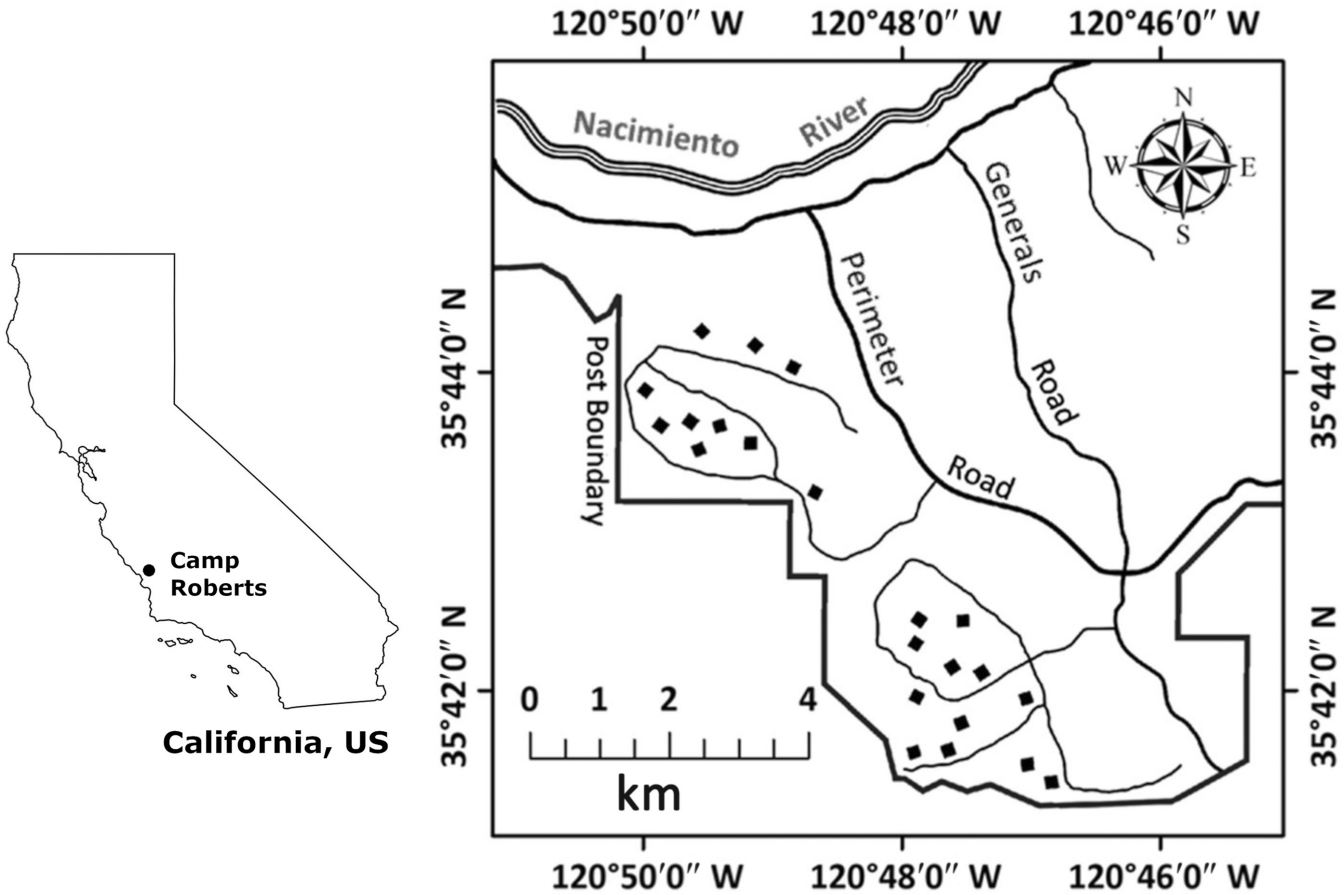
Study area at the National Guard Post, Camp Roberts, California, USA. The left panel shows the location of the study area (black, closed circle) within California. The right panel identifies the locations of the twenty‐two 1.1‐ha trapping grids (solid squares) where trapping occurred from 1997 to 2013.

Our study was carried out from 1997 to 2013 at the Army National Guard Post, Camp Roberts, in coastal‐central California, USA (Figure 1). Covering 8000 ha, the study area was a mosaic of grassland, chaparral, and woodland. The woodlands consisted of pure stands of blue oak (*Quercus douglasii*) with scattered buck brush (*Ceonothus cuneatus*) shrubs. The ground layer was dominated by Mediterranean annual grasses, predominantly *Avena* and *Bromus* spp., with scattered native bunch grasses (*Nassella* and *Festuca* spp.). In the more mesic areas, mixed stands of blue oak and coast live oak (*Quercus agrifolia*) occurred with a diverse shrub understory of toyon (*Heteromeles arbutifolia*), California coffee berry (*Frangula californica*), spiny red berry (*Rhamnus crocea*), buck brush (*Ceanothus cuneatus*), and bigberry manzanita (*Arctostaphylos glauca*). Grass cover was sparser than in the blue oak stands and intermixed with a diverse assemblage of native and exotic forbs, including wild peony (*Paeonia californica*), hummingbird sage (*Salvia spathacea*), deerweed (*Lotus scoparius*), and miner's lettuce (*Claytonia perfoliata*). Patches of poison oak (*Toxicodendron diversilobum*) occurred throughout the study area. Within these vegetation communities, the main suspected predators of small mammals were several species of owls: the barn owl (*Tyto alba*), great horned owl (*Bubo virginianus*), and California spotted owl (*Strix occidentalis occidentalis*). Snake species included the northern Pacific rattlesnake (*Crotalus oreganus oreganus*), Pacific gopher snake (*Pituophis catenifer catenifer*), and California kingsnake (*Lampropeltis californiae*). Large mammalian predators that frequented the study area were the mountain lion (*Puma concolor*), bobcat (*Lynx rufus*), coyote (*Canis latrans*), and gray fox (*Urocyon cinereoargenteus*).

The last wildfire within the study occurred in 1953. During the study, there was no disturbance by military activities. Public access to Camp Roberts was restricted to hunting for California quail (*Callipepla californica*), black‐tailed deer (*Odocoileus hemionus*), feral pigs (*Sus scrofa*), and wild turkeys (*Meleagris gallopavo*) during abbreviated seasons within the statewide hunting seasons.

### 2.2 Field methods

Between 1997 and 2013, we trapped small mammals twice annually (May and October) using Sherman live traps (3 x 3.5 x 30 cm; H.B. Sherman Traps, Inc.) for three trap nights on grids with 15‐m spacing (64 traps per grid). During spring 1997 to spring 2013, we trapped on 22 grids (33 sampling occasions) for 139,392 trap nights (22 grids * 64 traps/grid * 3 nights/grid * 33 sampling occasions = 139,392 trap nights). In fall 2013, 21 of the 22 grids were trapped (4032 trap nights). During the 17 years of study, we trapped during 34 sampling occasions and for 143,424 trap nights. At each capture, we recorded the capture location, and the species and sex of each animal. At first capture, we tagged animals in the right ear with a Monel 1.00S animal tag (National Band and Tag Co., Newport, Kentucky). We released all animals at the location of capture. Trapping and handling of animals followed the guidelines from the University of California, Berkeley, Institutional Animal Care and Use Committee (UC Berkeley Animal Care and USE Committee Permit R‐166), and the guidelines of the American Society of Mammalogists (Sikes & The Animal Care and Use Committee of the American Society of Mammalogists, 2016).

Our data collection protocol followed a Pollock's robust design approach (Pollock, 1982), with each season representing a primary sampling occasion, and each night of trapping within a season representing a secondary sampling occasion. Thus, our field sampling consisted of 34 sampling occasions (primary occasions), and secondary occasions consisting of 102 nights during which we trapped on each of the 22 trapping grids (34 primary trapping occasions and 3 nights/primary occasion; 34 x 3 = 102 secondary occasions). We assumed that the occupancy state of the small mammals was closed to changes during each secondary occasion within seasons, and open to changes in occupancy state from one primary occasion to another (MacKenzie et al., 2003, 2017).

### 2.3 Detection history for dynamic occupancy

In traditional dynamic occupancy (DO) analyses, each site (i.e., spatial unit) appears as a row and each sampling occasion appears as a column in the detection‐history matrix. For our application, however, we are interested in estimating the probability that each small mammal species from our pool of regional species is present on our study area. Hence, each species appeared as a row, and each sampling occasion as a column in the detection‐history matrix. During our study, 10 species of small mammals were captured and entered into our database. An additional two species (Californian ground squirrel *Otospermophilus beecheyi* and Botta's pocket gopher *Thomamys bottae*) were captured at least once, but the captures were not recorded. For these two species, we randomly assigned primary and secondary sampling occasions for when they might have been captured.

It is rare that all species (or all individuals of a species) are captured or detected during surveys of natural animal populations (Boulinier et al., 2001). Using the species range maps produced by the California Wildlife Habitat Relationships System (https://wildlife.ca.gov/Data/CWHR/Life‐History‐and‐Range), we determined that 18 additional species of small mammals could have been present on our study area but were not detected during our study (see Appendix S1: Table S1 for a complete species list). One or more of these species might have been present on our study area, but we had no way of knowing how many or which species were actually present but not captured. Thus, we augmented our detection history data by null detection histories (i.e., detection histories consisting of all zeroes) for these 18 species to indicate the possibility that these species might have been present but were missed. These augmented null detection‐history data permit estimation of ψ for the species that were potentially present but not detected (Kéry et al., 2009; MacKenzie et al., 2017). Thus, our detection‐history data matrix consisted of 30 rows (for 12 species that were detected at least once, and 18 species that may have been present but were not detected during our study), and 102 columns (for the 102 secondary sampling occasions).

### 2.4 Covariates

Current or past rainfall is known to influence survival and recruitment of small mammals in our study area (Srivathsa et al., 2019; Tietje et al., 2018), so we hypothesized that current total seasonal rainfall (sum of the daily rainfall during a season) or past total seasonal rainfall (sum of the daily rainfall lagged one and two seasons) would positively influence colonization (γ) and negatively influence extinction (ϵ). Regional climatic phenomena such as El Niño can potentially influence the DO parameters, so we also tested for the influence of the Oceanic Niño Index (ONI) on γ and ϵ. We obtained rainfall data for the years 1997 to 2013 from National Oceanic and Atmospheric Administration (2016) weather data recorded at the Paso Robles Municipal Airport, Paso Robles, California, ~11 km from the study area. We acquired monthly values of the Oceanic Niño Index (ONI) from the National Oceanographic and Atmospheric Agency Climate Prediction Center (https://www.ncei.noaa.gov/cdo‐web/). We also allowed γ and ϵ to vary between the dry (May–September) and wet (October–April) seasons, with the expectation that colonization would be higher and extinction probability lower during the wet season compared to the dry season. Furthermore, capture probability (*p)* and occupancy (ψ) can differ between commonly detected species and those that were rarely or never detected; thus, we also included species‐detection status as covariates for *p* and ψ. Species that were captured ≥1000 times during this study were considered commonly detected species (coded “1”), whereas those that were captured <1000 times or never captured during the study period were considered rarely detected species (coded “0”).

### 2.5 Data analysis

We analyzed the detection‐history data using the DO models (MacKenzie et al., 2003, 2017) to estimate the probabilities of detection (*p*), occupancy (ψ), and probabilities of time or season‐specific local extinction (ϵ_*t*_), and colonization (γ_*t*_). The occupancy probabilities for second and subsequent primary sampling occasions are then calculated recursively as represented in MacKenzie et al. (2003, 2017):

$$\psi_{t+1}=\psi_{t}\;\left(1-\epsilon_{t}\right)+\left(1-\psi_{t}\right)\;\gamma_{t}$$

where ψ_*t*_ and ψ_*t* + 1_ indicate the probability that the species from our list is present in the study area in two successive seasons, and γ_*t*_ and ϵ_*t*_ represent the colonization and extinction probability, respectively. Specifically, ψ_*t*_ in this context represents the fraction of the species in our regional species pool that was present in our study area at time *t*; γ_*t*_ represents the probability that a species that is absent on our study area at time *t* occupies the study area at time *t* + 1; and ϵ_*t*_ represents the probability that a species that is present on our study area at time *t* is absent from our study area at time *t* + 1 (MacKenzie et al., 2017).

Local species richness (SR) is determined by the species pool in the larger region (Cornell & Lawton, 1992). Species detected in any local area represents only a fraction of the species pool in the larger region, as some species may not be present in the local area or, if present, may go undetected. Thus, the local species richness, which also includes the species missed, can be calculated as the product of the number of species in the pool and the estimated fraction of the species pool present in the site as represented by:

SR=ψK

where ψ is the probability that species from our list is present on the site, SR is the number of species locally present, and *K* is the regional pool of species (Boulinier et al., 2001; Nichols et al., 2006). When the probability of each species being present is different, SR can be written as:

$$\mathrm{SR}=\sum_{s=1}^{n}\psi_{s}$$

where *n* is the possible number of species present in the area and ψ_s_ is the unconditional occupancy probability for each species (MacKenzie et al., 2003, 2017). Variance of ψ and species richness was estimated using the delta method (MacKenzie et al., 2017).

We implemented the DO model using program PRESENCE via the RPresence package (Hines, 2006; Hines & MacKenzie, 2015) for program R (R Development Core Team, 2022). We fit models using the function “occMod” based on our a priori hypotheses about seasonal variation in model parameters and covariate effects. To keep the number of models to a reasonable level, we first ran a set of models to determine an appropriate model structure for the detection probability, *p*. We hypothesized that *p* would differ between species that were commonly detected and those that were rarely detected or undetected and may differ across primary as well as secondary sampling occasions. These preliminary analyses revealed that models that allowed *p* to differ between commonly detected and rarely detected species was better supported than models that constrained *p* to be constant; thus, we used this model structure for *p* in all subsequent analyses. Next, we tested for the seasonal variation and temporal trends in γ and ϵ, as well as the singular effects of total seasonal rainfall (current and past), and the El Niño index. Finally, we tested for the influence of seasonal rainfall (current and past) and El Niño additively and interactively with season (dry or wet season).

We used an information‐theoretic approach using the Akaike Information Criterion (AIC) values for model selection and statistical inference (Burnham & Anderson, 2002). Covariate effects on the parameters of interest were assessed based on the differences in AIC values between models with and without the covariate, and by checking to see if 95% CIs for the regression coefficient(s) overlapped zero. When the top model could not estimate a parameter(s) of interest, estimates from the next best model were used. Species richness and associated confidence intervals were calculated by model averaging estimates from the top 10 models after excluding the top model (Appendix S1: Table S2; model no. 2–11). Unless otherwise indicated, we report mean parameter estimates ±1 SE.

For five species of small mammals with adequate sample size (California mouse [*Peromyscus californicus*], big‐eared woodrat [*Neotoma macrotis*], California pocket mouse [*Chaetodipus californicus*], brush mouse [*Peromyscus boylii*], and pinyon mouse [*Peromyscus truei*]), we used the superpopulation (or POPAN) capture‐mark‐recapture (CMR) modeling approach (Schwarz & Arnason, 1996; Williams et al., 2002) to estimate time‐specific population size and other demographic parameters. Details of CMR analyses are presented in Appendix S2.

## 3 RESULTS

### 3.1 Small mammal community composition

**FIGURE 2:**
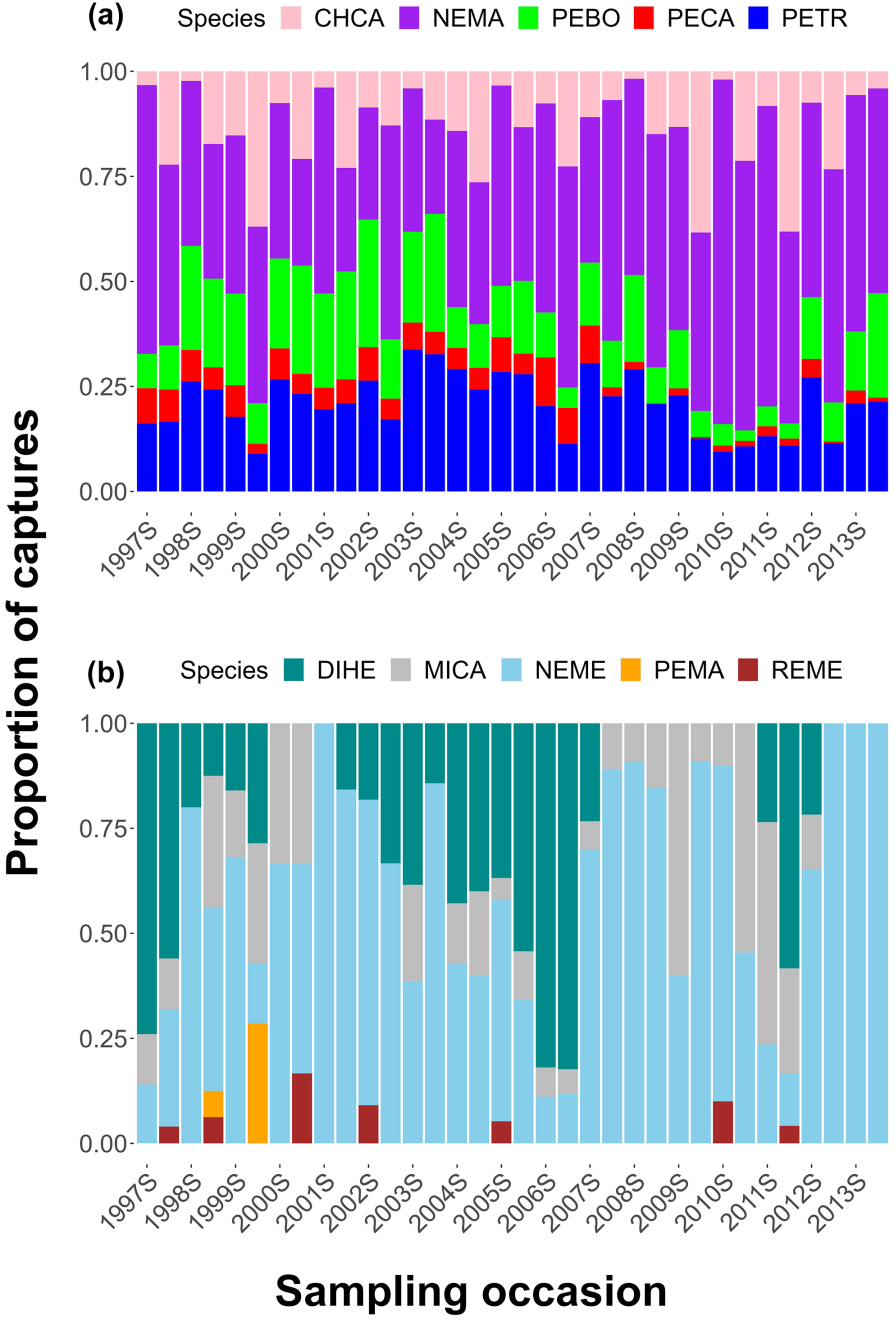
Proportion of small mammal captures during each year and season of study, 1997–2013, at Camp Roberts, California, USA, for species with ≥1000 (a) and <1000 captures (b). Captured species were: big‐eared woodrat (NEMA), brush mouse (PEBO), California mouse (PECA), California pocket mouse (CHCA), California vole (MICA), deer mouse (PEMA), Western harvest mouse (REME), Heermann's kangaroo rat (DIHE), Merriam's chipmunk (NEME), and pinyon mouse (PETR). The two species with <10 captures overall (California ground squirrel and Botta's pocket gopher) are not included in these plots. “S” in the year labels on the x‐axis represents the spring season.

Our sampling effort of 143,424 trap nights resulted in 45,174 captures of 12 small mammal species. The big‐eared woodrat was the most commonly captured species (26,167 captures; 57.93% of all captures) followed by pinyon mouse (7938 captures), brush mouse (4326 captures), California pocket mouse (3649 captures), and California mouse (2176 captures). The big‐eared woodrat represented 22% (2003 fall) to 82% (2010 spring) of total captures. The pinyon mouse and brush mouse were the two other frequently captured species, representing 9.65% (2010 spring) to 36.89% (2003 fall), and 3.17% (2010 fall) to 33.33% (2001 fall), respectively, of total captures (Figure 2). Four species, Heermann’s kangaroo rat (*Dipodomys heermanni*), California ground squirrel, Botta's pocket gopher, and Western harvest mouse (*Reithrodontomys megalotis*) were rarely captured. Marginally more captures occurred during spring (23,832; 53%) than fall (21,342; 47%). Five species (big‐eared woodrat, brush mouse, pinyon mouse, Merriam's chipmunk (*Neotamias merriami*), and California pocket mouse) were captured in every season, while three species were captured only in seven seasons or fewer (California ground squirrel, Heermann's kangaroo rat, and Western harvest mouse).

**FIGURE 3:**
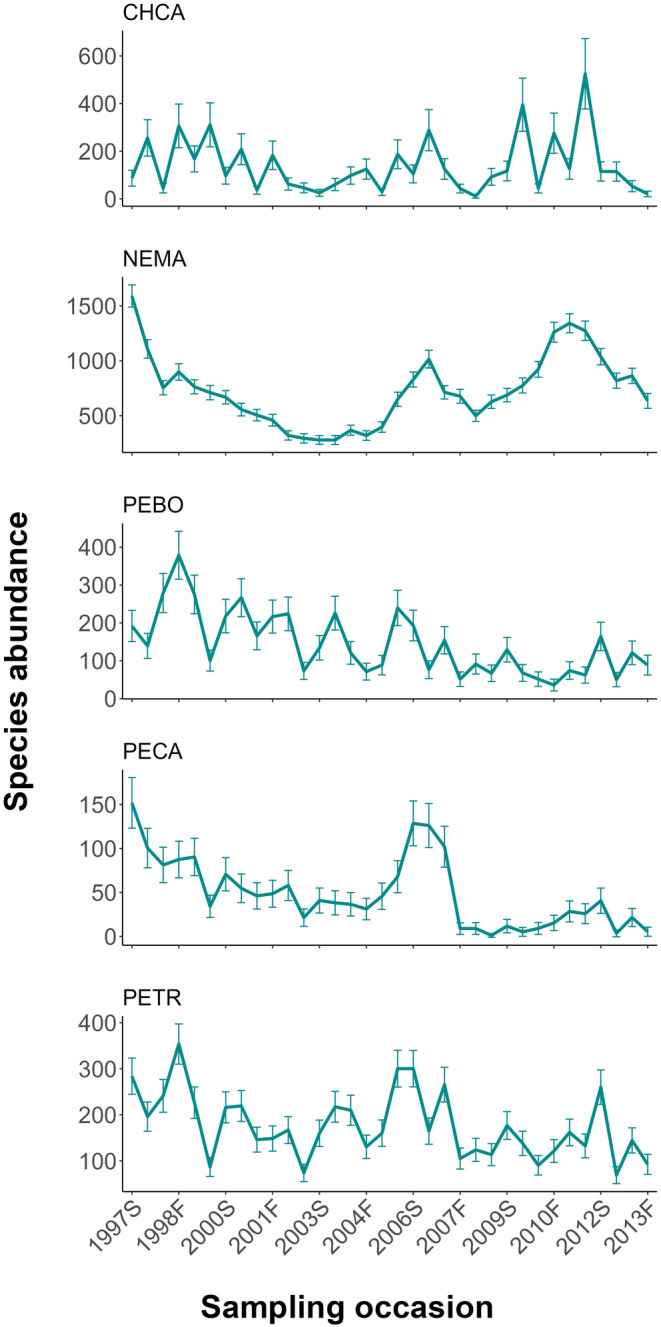
Estimated population size (solid line ± 1 SE) for the five most abundant small mammal species at Camp Roberts, California, USA, 1997–2013. These species are big‐eared woodrat (NEMA), pinyon mouse (PETR), brush mouse (PEBO), California mouse (PECA) and California pocket mouse (CHCA). “S” and “F” in the year labels on the x‐axis represent the spring and fall seasons.

**FIGURE 4:**
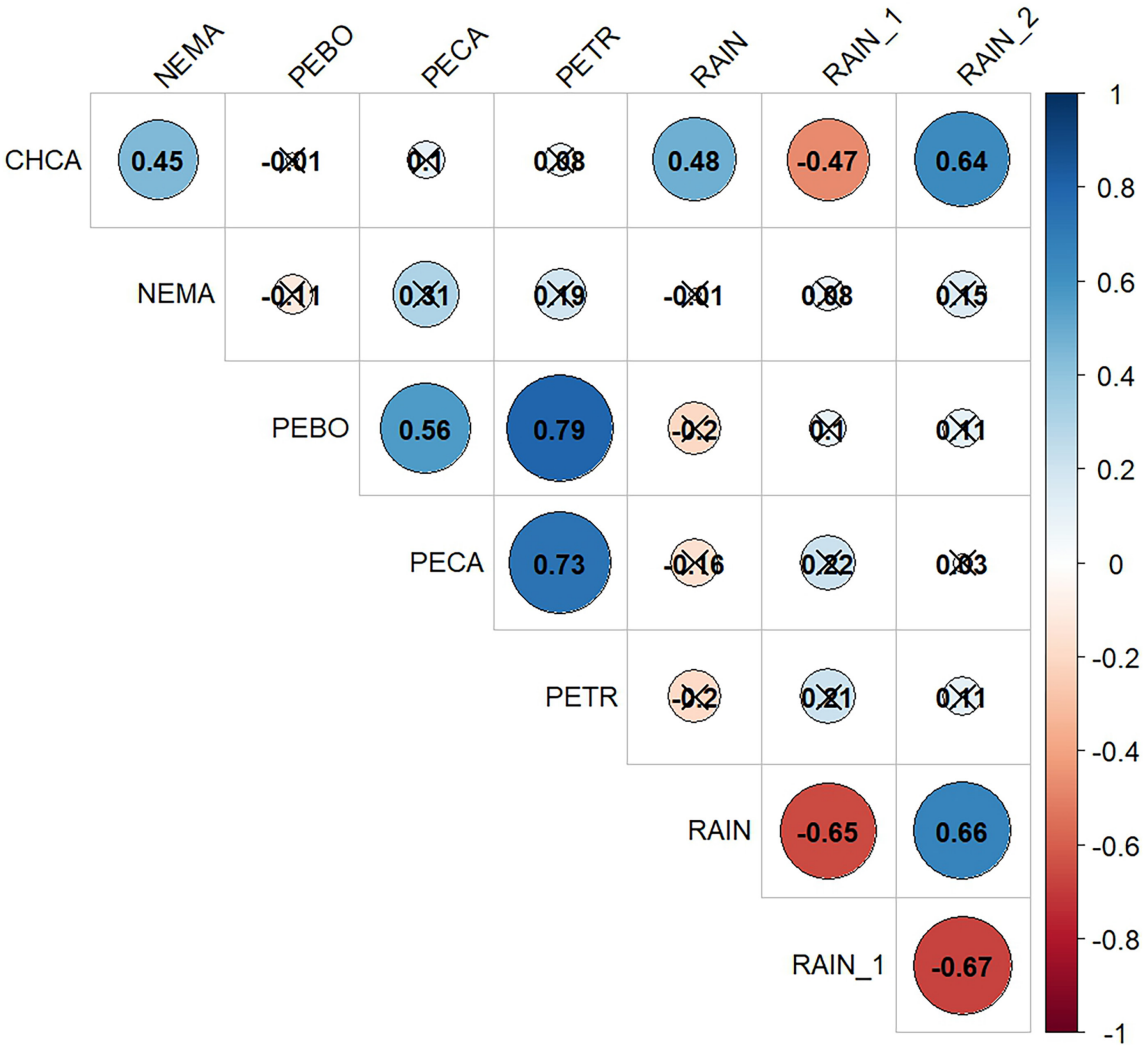
Pearson's correlation coefficients for species abundance and RAIN (total seasonal rainfall), RAIN_1 (total seasonal rainfall lagged one season), and RAIN_2 (total seasonal rainfall lagged two seasons) of the five most commonly captured small mammal species (big‐eared woodrat [NEMA], pinyon mouse [PETR], brush mouse [PEBO], California mouse [PECA], and California pocket mouse [CHCA]). Positive and negative correlations are indicated by blue and red colors, respectively, and darkness indicates the strength of correlation. Correlations that are not significant at *p* = .05 are indicated by crossed circles.

The absolute abundance of big‐eared woodrats often exceeded the absolute abundance of the next three most abundant species combined (Figure 3). Populations of all five small mammal species exhibited substantial seasonal and annual fluctuations without apparent temporal trends, except that brush mouse, pinyon mouse, and California mouse populations experienced slight declines during the study period. California pocket mouse abundance was positively correlated with total seasonal rainfall (*r* = .484, *p* = .004) and total seasonal rainfall lagged two seasons (*r* = .635, *p* < .001), but negatively correlated with total seasonal rainfall lagged one season (*r* = −.473, *p* = .005; Figure 4).

### 3.2 Dynamic occupancy parameters and species richness

**FIGURE 5:**
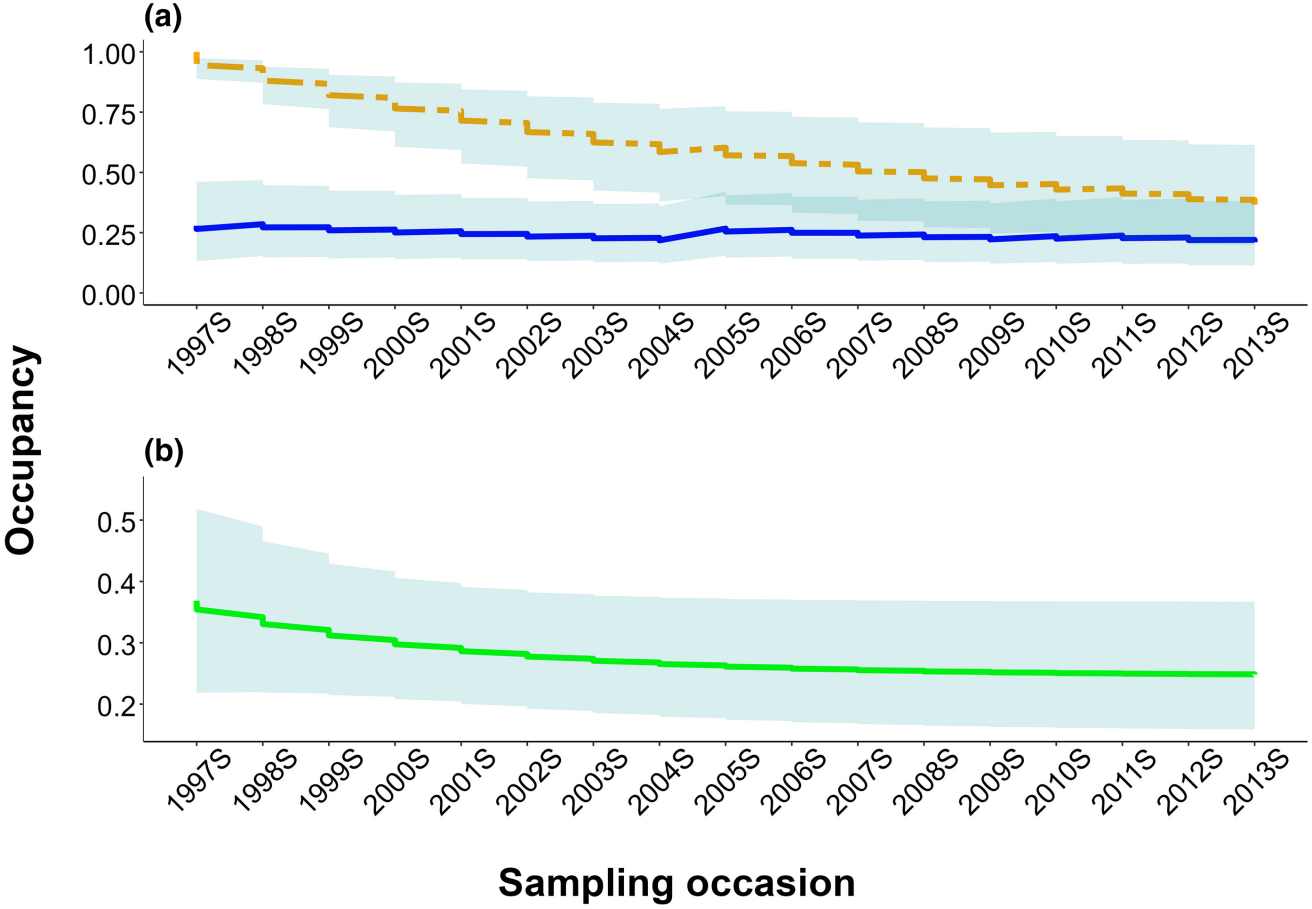
(a) Community occupancy probability (±95% confidence interval) of commonly detected species (orange dashed line) and rarely detected (or undetected) species (blue line) estimated based on model 1, Table 1C at Camp Roberts, California, USA, 1997–2013. (b) Overall community occupancy probability (± 95% confidence interval) of small mammals based on the constant parameter dynamic occupancy model (model 3, Table 1A). ”S” in the year labels on the x‐axis represents the spring season.

Based on the constant parameter DO model, parameter estimates ± SE were: initial occupancy probability $\left(\psi \right)=0.369\pm 0.088$, detection probability (*p*) = 0.852 ± 0.015, local colonization $\left(\gamma \right)=0.029\pm 0.007$, and extinction probability $\left(\epsilon \right)=0.088\pm 0.020$. However, the most parsimonious model (Model 1, Table 1B) suggested that *p* differed between commonly detected species and those that were rarely or never detected (*p*_common_ = 0.994 ± 0.003, *p*_rare_ = 0.546 ± 0.027), γ was lower during the dry than the wet season ($\gamma_{\mathrm{dry}}$ = 3.615e‐12 ± 1.436e‐07; $\gamma_{\mathrm{wet}}$ = 0.019 ± 0.008); and ϵ was low and quite stable during the study period (0.037 ± 0.012). However, this model could not estimate standard error for ψ for the frequently detected species group; consequently, this model could not be used to estimate time‐specific ψ. Thus, we used another model that received similar support (Model 1, Table 1C) to estimate time‐specific ψ. Based on this model, ψ was much higher for commonly detected species than for rarely detected species (ψ_common_ = 1.000 ± 0.000, ψ_rare_ = 0.281 ± 0.091). Seasonally, ϵ was higher during the dry season than during the wet season (ϵ_dry_ = 0.055 ± 0.021; ϵ_wet_ = 0.016 ± 0.014). Small mammal occupancy decreased over time from 0.369 ± 0.088 in 1997 to 0.248 ± 0.054 in 2013 (Figure 5a); however, this decline was steeper for the commonly detected species than for the rarely detected species (Figure 5b). Likewise, small mammal species richness declined from 11.943 ± 0.461 species in 1997 to 7.185 ± 0.425 species in 2013 (Figure 7).

**TABLE 1:** Model selection results for the dynamic occupancy models fitted to estimate initial occupancy (ψ), detection probability (p), probability of local colonization (γ) and extinction (ϵ) for small mammals on our study area at Camp Roberts, California. Note: See the Methods section and the Note below TABLE 1 for definitions of the covariates used in the models‐selection results lists in parts A, B, and C of the Table.

		*K*	AIC	ΔAIC	Weight
A	Detection models				
1	ψ(.) p(status) γ(.) ϵ(.)	5	856.76	0.000	0.998
2	ψ(.) p(SEASON + status) γ(.) ϵ(.)	38	869.23	12.470	0.002
3	ψ(.) p(.) γ(.) ϵ(.)	6	1114.55	257.790	0.000
4	ψ(.) p(SURVEY) γ(.) ϵ(.)	6	1117.99	261.230	0.000
B	Top four models without climatic covariates				
1	ψ(status) p(status) γ(season) ϵ(.)^**#**^	7	844.596	0.000	0.144
2	ψ(status) p(status) γ(.) ϵ(season)	7	848.136	3.540	0.025
3	ψ(.) p(status) γ(season) ϵ(season)	7	852.926	8.330	0.002
4	ψ(.) p(status) γ(.) ϵ(.)	4	857.766	12.170	0.000
C	Top four models with climatic covariates				
1	ψ(status) p(status) γ(RF) ϵ(season)	8	844.746	0.150	0.133
2	ψ(status) p(status) γ(RF) ϵ(.)	7	844.886	0.290	0.124
3	ψ(status) p(status) γ(RF) ϵ(El Niño)	8	844.956	0.360	0.120
4	ψ(status) p(status) γ(season + RF) ϵ(.)	8	845.196	0.600	0.106

*Note*: Table 1 presents the top four models: (A) to determine the best model structure for the detection probability, *p*; (B) to estimate γ and ϵ without including climatic covariates, and (C) to estimate γ and ϵ incorporating the climatic covariates. The covariates considered are: SEASON (an inbuilt covariate in the program Rpresence that corresponds to the 34 primary sampling occasions, that is, May and October trapping); SURVEY (an inbuilt covariate in Rpresence that represents the secondary sampling occasions, that is, the 102 days of trapping within the 34 primary sampling occasions; status (the number of captures, that is, <1000 total captures or ≥1000 total captures); season (spring or fall); RF (total seasonal rainfall); RF_1lag_ (total seasonal rainfall lagged one season); RF_2lag_ (total seasonal rainfall lagged two seasons); and El Niño. For each model, the number of parameters (*K*), Akaike’s information criterion (AIC), ΔAIC, and Weight are presented. A dot (.) indicates a constant parameter and a plus sign (+) indicates an additive effect. The overall top model is indicated by #.

**FIGURE 6:**
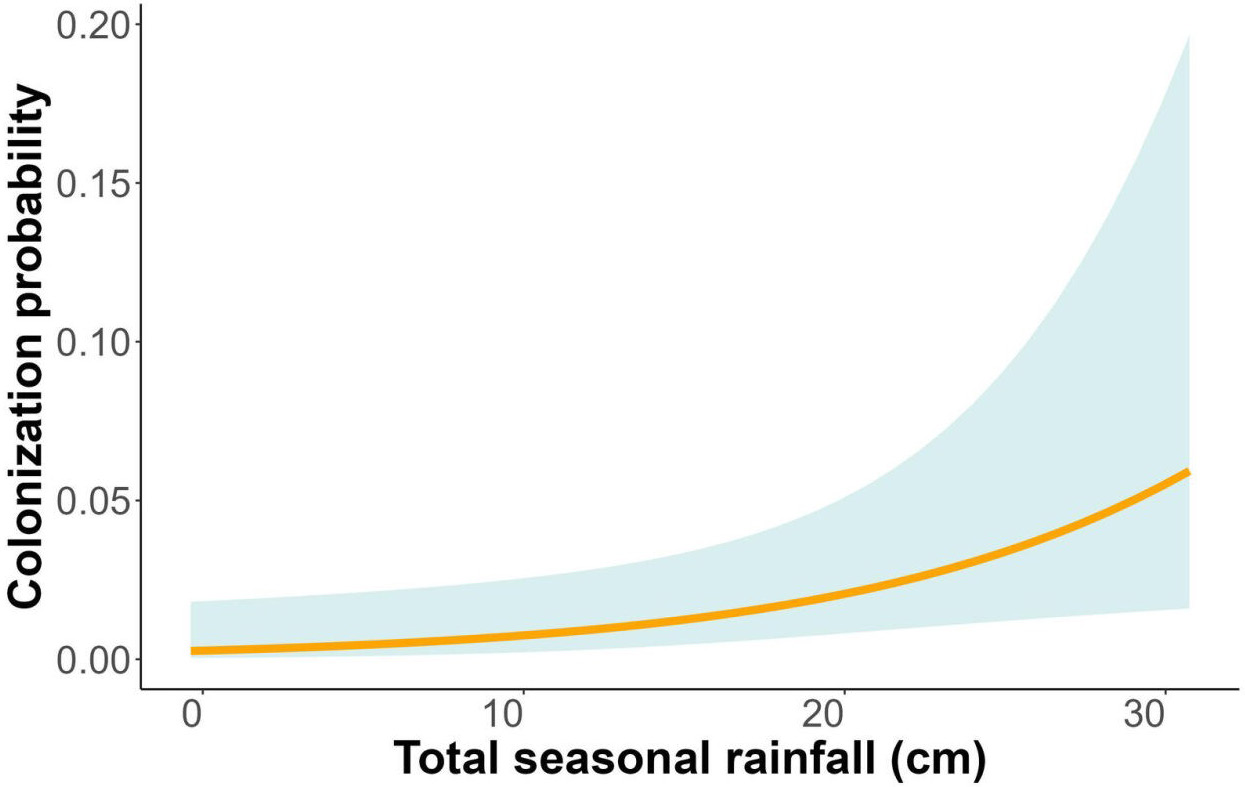
Small mammal colonization probability as a function of total seasonal rainfall at Camp Roberts, California, USA, 1997–2013. This relationship is based on model 1, Table 1C.

Inclusion of climatic covariates considered in this study did not substantially improve model parsimony (Table 1C). Nonetheless, we examined the effect size for each climatic covariate (singularly, additively, or interactively with season) based on regression coefficients (slope parameter, β and 95% CI) of the top model containing a particular covariate. We found evidence for a positive influence of total seasonal rainfall on γ (β = 0.859 ± 0.371; 95% CI = 0.132–1.587; Figure 6). Other climatic covariates (total seasonal rainfall lagged one season, total seasonal rainfall lagged two seasons, and El Niño) considered in our study did not influence the DO parameters (γ and ϵ); 95% CI for β parameters relating climatic covariates to γ and ϵ straddled zero.

## 4 DISCUSSION

Across continents, community dynamics of small mammals in semiarid environments is strongly influenced by rainfall patterns (Kelt et al., 1996; Meserve et al., 2011). Previous studies from our study area have provided evidence that populations of some small mammal species were affected by climatic factors such as rainfall and temperature (Polyakov et al., 2021; Rolland et al., 2021; Srivathsa et al., 2019; Tietje et al., 2018). Given these findings and the prediction that coastal California will experience a more variable climate and greater frequency and intensity of drought events (Hayhoe et al., 2004; Moser et al., 2012; Rapacciuolo et al., 2014), it is of ecological and conservation importance to ask how predicted climatic changes might influence the dynamics of a small mammal community. Here, we used a 17‐year small mammal trapping dataset to understand whether or to what extent the abundance, composition, and richness of the small mammal community inhabiting a coastal California oak woodland changed during the study period, and to evaluate the influence of rainfall on species richness and the dynamic occupancy (DO) parameters.

During this study period, 12 species of small mammals were captured at least once, but the number of species captured varied from a minimum of six species in the fall of 2008 and 2013 to a maximum of 11 species in the spring of 1997. The big‐eared woodrat was overwhelmingly the most dominant species throughout the study in terms of abundance. Big‐eared woodrats achieved relatively high densities in 1997, 2007, and 2011; they occurred in low numbers during 2000–2004. On average, the abundance of the California mouse, brush mouse, and pinyon mouse declined during this study, whereas all of the small mammal populations showed seasonal and/or random annual fluctuations. Seasonal fluctuations in California pocket mouse abundance was because they remain underground and unavailable for capture during the spring season. Heermann's kangaroo rat and California ground squirrel were infrequently captured before 2001 and were not captured after that year. We are not sure whether they went locally extinct or simply did not get captured in later years. We postulated that the abundance and potential dominance of the big‐eared woodrat would negatively influence the abundance of other species (Previtali et al., 2009); however, there was no evidence for this (*p* > .05). The abundance of pinyon mouse showed strong positive correlation with the abundances of brush mouse (*r* = .790, *p* < .001) and California mouse (*r* = .734, *p* < .001), which might warrant further attention. Also, despite the positive association of rainfall with the γ of small mammals, except for the California pocket mouse, the abundance of individual species did not significantly correlate with rainfall.

**FIGURE 7:**
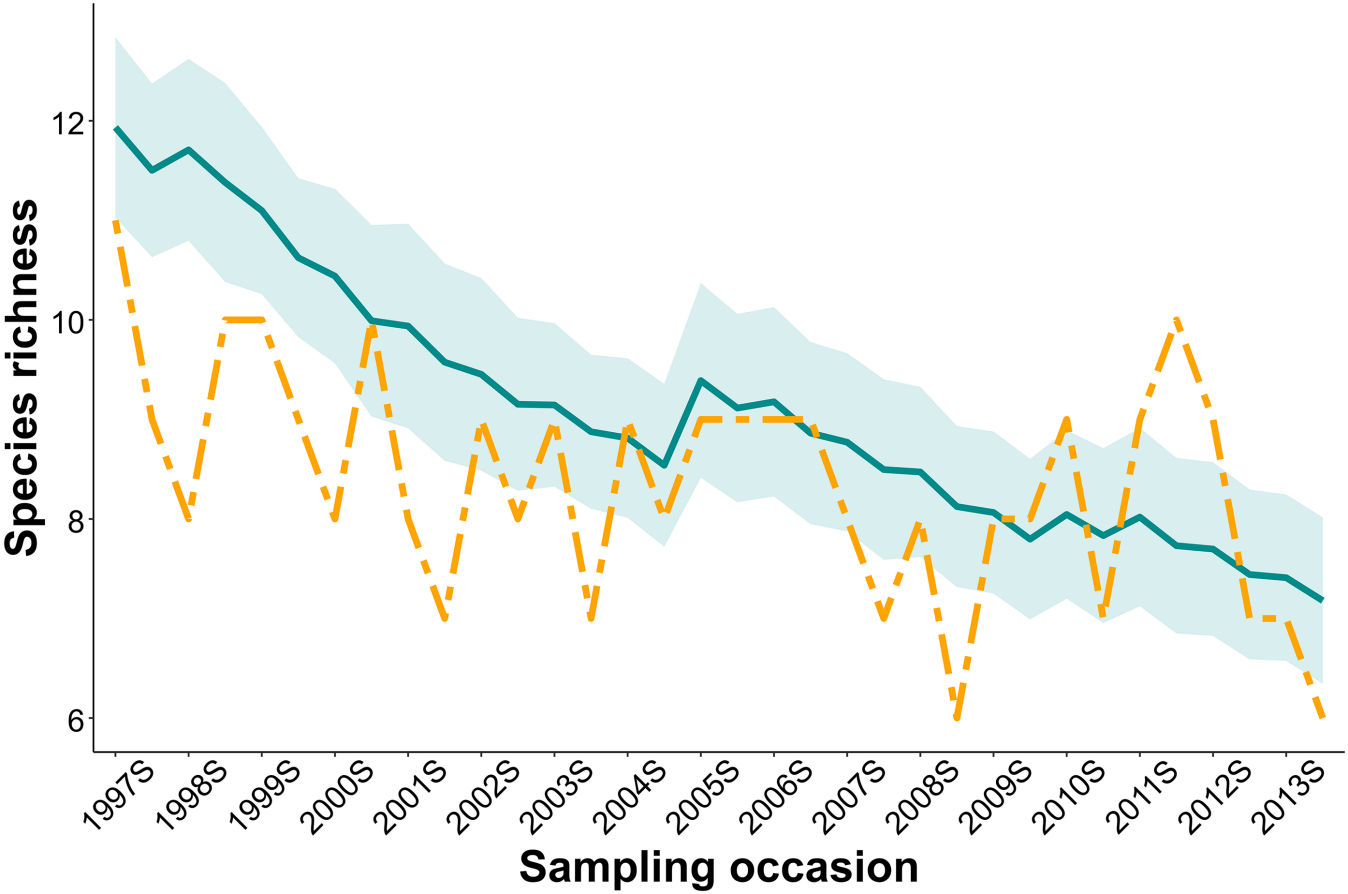
Model averaged species richness (±95% confidence interval) of small mammals (blue line) and the number of species detected in each survey season (orange dashed line) on our study area at Camp Roberts, California, USA, 1997–2013. “S” in the year labels on the x‐axis represents the spring season.

The DO model revealed that extinction probability (ϵ) was higher than colonization probability (γ) at Camp Roberts. Being a semiarid area, we expected γ to be higher and ϵ to be lower during the wet season compared to dry season at Camp Roberts. Consistent with our hypothesis, estimates of γ were substantially higher during the wet season, with near zero γ during the dry season. This higher γ during winter is possibly facilitated by the positive influence of rainfall on the survival and recruitment of small mammals, which is well documented in arid areas (Meserve et al., 2011; Previtali et al., 2009). In contrast, and also consistent with our hypothesis, ϵ was about four times higher during the dry than the wet season. These estimated dynamic community occupancy parameters imply that small mammal species richness on our study area would decline over time, because γ is near zero during the dry season, whereas ϵ is nonzero for both the dry and wet seasons. As a result, ϵ is not balanced by γ, which would cause both small mammal occupancy probability and species richness to decline over time. Indeed, that is precisely what we found (Figures 5 and 7). We observed a ~ 33% decrease in small mammal occupancy during our study period, from 0.369 ± 0.088 in spring 1997 to 0.248 ± 0.054 fall 2013; the rate of decline was much higher (~62%) for common species than for rarely detected species (~25%).

In addition to the higher extinction and lower colonizaion rates that occurred during our study, species richness decreased from 11.943 ± 0.461 species in spring 1997 to 7.185 ± 0.425 species in fall 2013, a striking decline of 40% during the study period. Gillespie et al. (2008) also documented decreased abundances and even local extinction of two small mammal species, namely the California kangaroo rat (*Dipodomys californicus)* and the dusky‐footed woodrat (*Neotoma fuscipes*) in northern California, but found no clear evidence that these extinction events were triggered by climatic variables. However, evidence suggests that local extinctions do occur and that species richness might decrease under the scenario of changing climate (Thomas et al., 2006; Urban, 2015). This becomes especially critical in semiarid areas such as coastal‐central California where rainfall is an important determinant of primary production, species survival, and recruitment and thus species richness (Jin & Goulden, 2014; Meserve et al., 2011). A similar negative influence on small mammal populations, predictably with rarer species bearing the worst impacts, may occur in California where prolonged droughts are expected to be more frequent and intense (Cook et al., 2014; Dai, 2013; Moser et al., 2012).

Given our findings and those of other studies of small mammals in arid or semiarid environments (Farias et al., 2021; Kelt et al., 1996; Meserve et al., 2011) and predictions regarding the changing climatic patterns, it seems likely that small mammal communities inhabiting arid and semiarid environments will be negatively impacted in the foreseeable future. We note that our study was conducted in a relatively small area (8000 ha) in one location (coastal California) for a fairly short period of time (~17 years). Climatic influences on demography, population dynamics, and species richness can vary regionally. Studies at larger spatial and temporal scales in multiple locations would be essential to provide a comprehensive assessment of climatic influences on small mammal population and community dynamics under changing climatic conditions. Such an approach would also allow wildlife managers to detect changes in the mammalian community composition and species richness, and to implement conservation interventions in a timely fashion (Chaudhary et al., 2022; Crego et al., 2020).

## AUTHOR CONTRIBUTIONS

**Yadav P. Ghimirey:** Data curation (equal); formal analysis (equal); writing – original draft (equal); writing – review and editing (equal). **William D. Tietje:** Conceptualization (equal); funding acquisition (lead); project administration (lead); validation (equal); writing – review and editing (equal). **Anne Y. Polyakov:** Formal analysis (equal); writing – review and editing (equal). **James E. Hines:** Methodology (equal); validation (equal); writing – review and editing (equal). **Madan K. Oli:** Conceptualization (lead); data curation (equal); investigation (equal); methodology (equal); supervision (lead); validation (equal); writing – review and editing (equal).

## CONFLICT OF INTEREST STATEMENT

We have no competing interests.

### OPEN RESEARCH BADGES

This article has earned a Preregistered Research Designs badge for having a preregistered research design, available at [https://datadryad.org/stash/share/X_YsIVeKdAKEGsChqyKJaY4JzYQv‐Y1ILSbSF9jzg9M].

## Supporting information


Appendix S1.
Appendix S2.

## Data Availability

Data and R code used in this study are available from the Digital Object Identifier Foundation Repository: https://datadryad.org/stash/share/X_YsIVeKdAKEGsChqyKJaY4JzYQv‐Y1ILSbSF9jzg9M.
