# Supplementary material for: Decline in small mammal species richness in coastal‐central California, 1997–2013
Source: Ecol Evol. 2023 Dec 10;13(12):e10611. doi: 10.1002/ece3.10611 (PMC10711327; doi:10.1002/ece3.10611)
Supplement: Supplementary file 1 — Appendix S1. Appendix S2. [file ECE3-13-e10611-s001.docx]

## **Supporting Information 1**

**Table S1.** List of small mammals considered in the present study for population and community analysis at Camp Roberts, California. The first 12 species (highlighted in bold) were detected at least once during our 17-yr (1997 – 2013) live trapping study. The remaining (18 species) were identified as possibly present species in the study area based on California Wildlife Habitat Relationships range maps but not captured during our study.

| Species | Scientific Name |
| --- | --- |
| **Kangaroo rat** | ***Dipodomys heermanni*** |
| **California vole** | ***Microtus californicus*** |
| **Big-eared woodrat** | ***Neotoma macrotis*** |
| **Brush mouse** | ***Peromyscus boylii*** |
| **California mouse** | ***Peromyscus californicus*** |
| **Deer mice** | ***Peromyscus maniculatus*** |
| **Pinyon mouse** | ***Peromyscus truei*** |
| **Pocket mouse** | ***Chaetodipus californicus*** |
| **Harvest mouse** | ***Reithrodontomys megalotis*** |
| **Merriam's chipmunk** | ***Neotamias merriami*** |
| **Botta’s pocket gopher** | ***Thomomys bottae*** |
| **California ground squirrel** | ***Otospermophilus beecheyi*** |
| House mouse | *Mus musculus* |
| Southern broad-footed mole | *Scapanus latimanus* |
| Ornate shrew | *Sorex ornatus* |
| Trowbridge shrew | *Sorex trowbridgii* |
| Vagrant shrew | *Sorex vagrans* |
| Long-tailed weasel | *Neogale frenata* |
| Common muskrat | *Ondatra zibethicus* |
| Audubon's cottontail | *Sylvilagus audubonii* |
| Black-tailed jackrabbit | *Lepus californicus* |
| Brush rabbit | *Sylvilagus bachmani* |
| Fresno kangaroo rat | *Dipodomys nitratoides* |
| Giant kangaroo rat | *Dipodomys ingens* |
| Narrow-faced kangaroo rat | *Dipodomys venustus* |
| Nelson's antelope ground squirrel | *Ammospermophilus nelsoni* |
| San Joaquin pocket mouse | *Perognathus inornatus* |
| Southern grasshopper mouse | *Onychomys torridus* |
| Western grey squirrel | *Sciurus griseus* |
| Black rat | *Rattus rattus* |

**Figure S1.** The relationship between total seasonal rainfall and estimated species richness during (A) spring and (B) fall season at Camp Roberts, California, 1997-2013. The points represent species richness estimates each season, solid line represents predicted relationship between species richness and rainfall, whereas the gray band represents 95% confidence intervals. Note the difference in x-axis scale between the panels.


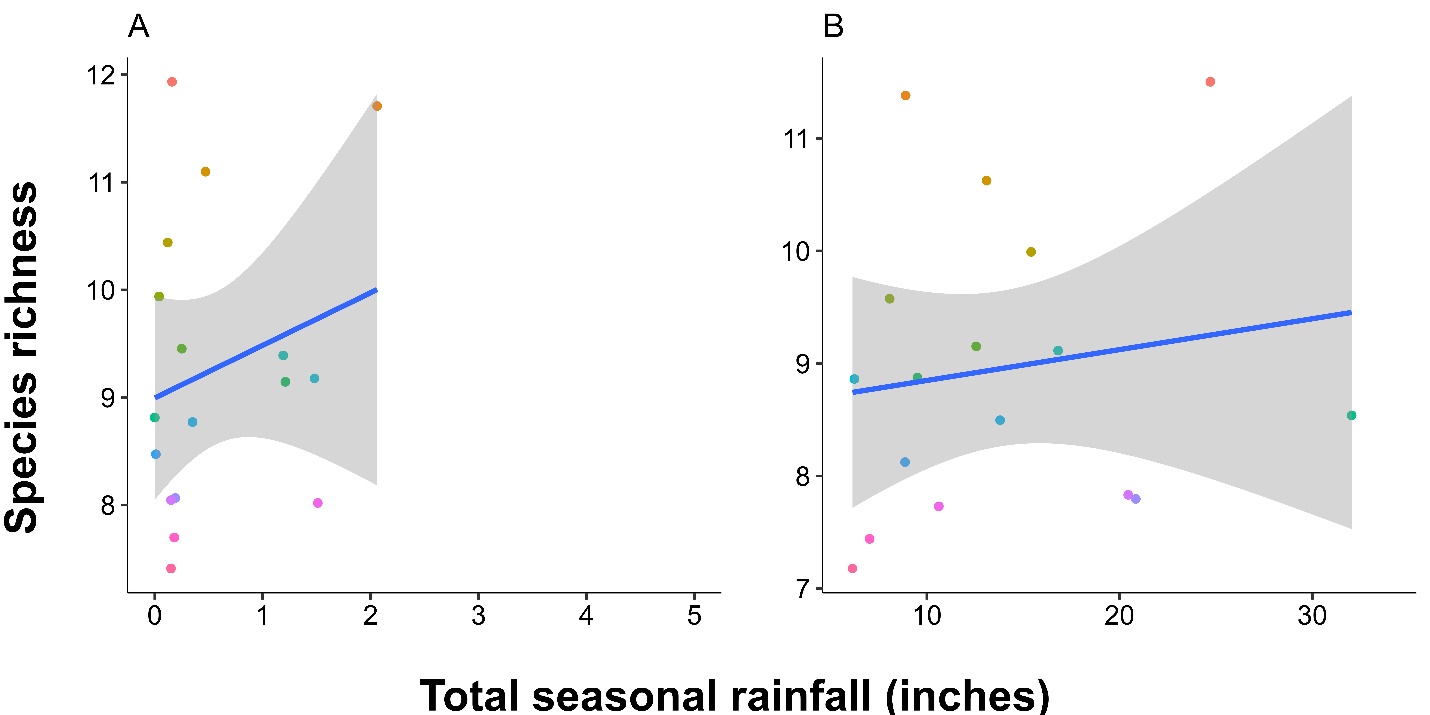


**Table S2.** A comparison of all the models fitted to assess the influence of species status (common or rare) on occupancy ($\psi$); species status, SEASON (each season of each year; an internally created covariate in RPresence), SURVEY (secondary survey during each season and year; an internally created covariate in RPresence) on detection (*p*); and Season (fall or spring), total seasonal rainfall (RF), total seasonal rainfall lagged one season (RF_1lag_), total seasonal rainfall lagged two seasons (RF_2lag_) and El Niño on local colonization ($\gamma$) and extinction probability ($\epsilon$) of small mammals in Camp Roberts, coastal-central California. A total of 52 models were fitted. The table presents Akaike information criterion (AIC), difference in AIC value from the top model (ΔAIC), model strength or probability (Weight) and number of parameters (K). A dot (.) indicates constant parameter, a plus (+) sign indicates additive effects and an asterisk sign (*) indicates additive as well as interactive effect of the covariates involved in the model.

| Model no. | Model | AIC | ΔAIC | Weight | K |
| --- | --- | --- | --- | --- | --- |
| 1 | $\psi$(status) $p$(status) $\gamma$(season) $\epsilon$(.) | 844.596 | 0.000 | 0.142 | 7 |
| 2 | $\psi$(status) $p$(status) $\gamma$(RF) $\epsilon$(season) | 844.746 | 0.150 | 0.132 | 8 |
| 3 | $\psi$(status) $p$(status) $\gamma$(RF) $\epsilon$(.) | 844.886 | 0.290 | 0.123 | 7 |
| 4 | $\psi$(status) $p$(status) $\gamma$(RF) $\epsilon$(El Niño) | 844.956 | 0.360 | 0.119 | 8 |
| 5 | $\psi$(status) $p$(status) $\gamma$(season+RF) $\epsilon$(.) | 845.196 | 0.600 | 0.105 | 8 |
| 6 | $\psi$(status) $p$(status) $\gamma$(El Niño+RF) $\epsilon$(.) | 846.386 | 1.790 | 0.058 | 8 |
| 7 | $\psi$(status) $p$(status) $\gamma$(RF+RF_2lag_) $\epsilon$(.) | 846.866 | 2.270 | 0.046 | 8 |
| 8 | $\psi$(status) $p$(status) $\gamma$(.)$\epsilon$(El Niño) | 847.196 | 2.600 | 0.039 | 7 |
| 9 | $\psi$(status) $p$(status) $\gamma$(RF*season) $\epsilon$(.) | 847.196 | 2.600 | 0.039 | 9 |
| 10 | $\psi$(status) $p$(status) $\gamma$(El Niño*RF) $\epsilon$(.) | 847.576 | 2.980 | 0.032 | 9 |
| 11 | $\psi$(status) $p$(status) $\gamma$(.)$\epsilon$(season) | 848.136 | 3.540 | 0.024 | 7 |
| 12 | $\psi$(status) $p$(status) $\gamma$(RF_2lag_*season) $\epsilon$(.) | 848.236 | 3.640 | 0.023 | 9 |
| 13 | $\psi$(status) $p$(status) $\gamma$(RF_2lag_) $\epsilon$(El Niño) | 848.366 | 3.770 | 0.022 | 8 |
| 14 | $\psi$(status) $p$(status) $\gamma$(RF_2lag_) $\epsilon$(season) | 848.816 | 4.220 | 0.017 | 8 |
| 15 | $\psi$(status) $p$(status) $\gamma$(El Niño) $\epsilon$(El Niño) | 849.146 | 4.550 | 0.015 | 8 |
| 16 | $\psi$(status) $p$(status) $\gamma$(.)$\epsilon$(El Niño*season) | 849.166 | 4.570 | 0.015 | 9 |
| 17 | $\psi$(status) $p$(status) $\gamma$(RF*season) $\epsilon$(El Niño*season) | 849.276 | 4.680 | 0.014 | 12 |
| 18 | $\psi$(status) $p$(status) $\gamma$(.)$\epsilon$(RF_1lag_) | 849.556 | 4.960 | 0.012 | 7 |
| 19 | $\psi$(status) $p$(status) $\gamma$(El Niño) $\epsilon$(.) | 849.836 | 5.240 | 0.010 | 7 |
| 20 | $\psi$(status) $p$(status) $\gamma$(El Niño) $\epsilon$(season) | 852.926 | 5.500 | 0.009 | 8 |
| 21 | $\psi$(.) $p$(status) $\gamma$(season) $\epsilon$(season) | 853.576 | 8.330 | 0.002 | 7 |
| 22 | $\psi$(.) $p$(status) $\gamma$(season) $\epsilon$(El Niño) |  | 8.980 | 0.002 | 5 |
| 23 | $\psi$(status) $p$(.)$\gamma$(season+RF_1lag_) $\epsilon$(.) | 1103.936 | 259.340 | 0.000 | 7 |
| 24 | $\psi$(status) $p$(.)$\gamma$(.)$\epsilon$(.) | 1104.736 | 260.140 | 0.000 | 5 |
| 25 | $\psi$(status) $p$(.)$\gamma$(.)$\epsilon$(RF*season) | 1104.816 | 260.220 | 0.000 | 8 |
| 26 | $\psi$(status) $p$(.)$\gamma$(RF_2lag_) $\epsilon$(RF) | 1105.176 | 260.580 | 0.000 | 7 |
| 27 | $\psi$(status) $p$(.)$\gamma$(.)$\epsilon$(El Niño*RF) | 1105.216 | 260.620 | 0.000 | 7 |
| 28 | $\psi$(status) $p$(.)$\gamma$(RF+RF_1lag_) $\epsilon$(.) | 1105.726 | 261.130 | 0.000 | 7 |
| 29 | $\psi$(status) $p$(.)$\gamma$(.)$\epsilon$(season+RF) | 1105.846 | 261.250 | 0.000 | 7 |
| 30 | $\psi$(status) $p$(.)$\gamma$(El Niño) $\epsilon$(RF) | 1105.896 | 261.300 | 0.000 | 7 |
| 31 | $\psi$(status) $p$(.)$\gamma$(.)$\epsilon$(RF+RF_1lag_) | 1105.906 | 261.310 | 0.000 | 7 |
| 32 | $\psi$(status) $p$(.)$\gamma$(RF_1lag_) $\epsilon$(RF) | 1105.926 | 261.330 | 0.000 | 7 |
| 33 | $\psi$(status) $p$(.)$\gamma$(.)$\epsilon$(RF+RF_2lag_) | 1105.936 | 261.340 | 0.000 | 7 |
| 34 | $\psi$(status) $p$(.)$\gamma$(.)$\epsilon$(season+RF_1lag_) | 1105.936 | 261.340 | 0.000 | 7 |
| 35 | $\psi$(status) $p$(.)$\gamma$(RF_1lag_) $\epsilon$(season) | 1106.116 | 261.520 | 0.000 | 7 |
| 36 | $\psi$(status) $p$(.)$\gamma$(El Niño*season) $\epsilon$(El Niño*season) | 1106.356 | 261.760 | 0.000 | 11 |
| 37 | $\psi$(status) $p$(.)$\gamma$(RF*season) $\epsilon$(RF*season) | 1106.976 | 262.380 | 0.000 | 11 |
| 38 | $\psi$(status) $p$(.)$\gamma$(RF_1lag_) $\epsilon$(RF_1lag_) | 1107.706 | 263.110 | 0.000 | 7 |
| 39 | $\psi$(status) $p$(.)$\gamma$(RF+season) $\epsilon$(RF+season) | 1107.986 | 263.390 | 0.000 | 9 |
| 40 | $\psi$(status) $p$(.)$\gamma$(El Niño*season) $\epsilon$(.) | 1108.746 | 264.150 | 0.000 | 8 |
| 41 | $\psi$(status) $p$(.)$\gamma$(El Niño*season) $\epsilon$(RF*season) | 1108.776 | 264.180 | 0.000 | 11 |
| 42 | $\psi$(.)$p$(.)$\gamma$(.)$\epsilon$(RF) | 1113.756 | 269.160 | 0.000 | 5 |
| 43 | $\psi$(.)$p$(.)$\gamma$(season) $\epsilon$(RF) | 1114.066 | 269.470 | 0.000 | 6 |
| 44 | $\psi$(.)$p$(.)$\gamma$(season) $\epsilon$(season) | 1114.276 | 269.680 | 0.000 | 6 |
| 45 | $\psi$(.)$p$(.)$\gamma$(.)$\epsilon$(.) | 1114.556 | 269.960 | 0.000 | 4 |
| 46 | $\psi$(.)$p$(.)$\gamma$(RF) $\epsilon$(.) | 1115.366 | 270.770 | 0.000 | 5 |
| 47 | $\psi$(.)$p$(.)$\gamma$(.)$\epsilon$(RF_1lag_) | 1115.566 | 270.970 | 0.000 | 5 |
| 48 | $\psi$(.)$p$(.)$\gamma$(.)$\epsilon$(El Niño) | 1115.596 | 271.000 | 0.000 | 5 |
| 49 | $\psi$(.)$p$(.)$\gamma$(RF_2lag_) $\epsilon$(.) | 1116.136 | 271.540 | 0.000 | 5 |
| 50 | $\psi$(.)$p$(.)$\gamma$(RF_1lag_) $\epsilon$(.) | 1116.456 | 271.860 | 0.000 | 5 |
| 51 | $\psi$(.)$p$(.)$\gamma$(El Niño) $\epsilon$(.) | 1116.556 | 271.960 | 0.000 | 5 |
| 52 | $\psi$(.) $p$(.)$\gamma$(RF_1lag_) $\epsilon$(El Niño) | 1117.556 | 272.960 | 0.000 | 6 |

##

## **Supporting Information 2**

**Capture-Mark-Recapture modeling of small mammal populations in Camp Roberts, coastal central California 1997 – 2013.**

We carried out small mammal live trapping in coastal central California between 1997 and 2013 and applied capture-mark-recapture (CMR) models to five species of mammals with sufficient data (big-eared woodrat (NEMA), California mouse (PECA), brush mouse (PEBO), pinyon mouse (PETR) and pocket mouse (CHCA)) to estimate demographic parameters, especially apparent survival probability. Because we were also interested in estimating abundance, we chose the superpopulation (or POPAN) model for our analyses (Williams et al. 2002).

We hypothesized that i) capture probability would differ between seasons and sexes, and vary across sampling occasions (time); ii) apparent survival probability and probability of entrance would vary over time, and differ between seasons and sexes, and both an additive and interactive effect of season and sex, in addition to sampling occasion; and iii) superpopulation size would differ between sexes.

**Methods**

Between 1997 and 2013, small mammals were trapped twice annually on grids with 15-m spacing, once each in the spring (mostly in May) and fall (mostly in October) seasons using Sherman live traps (3 × 3.5 × 30 cm; H.B. Sherman Traps, Inc., Tallahassee, Florida). During spring 1997 to spring 2013, we trapped on 22, 1.1-ha grids (33 primary trapping sessions) for 3 nights each session (22 grids * 64 traps/grid * 3 nights/grid * 33 sessions = 139,392 trap nights). In fall 2013, we trapped on 21 of the 22 grids (4,032 trap nights). During the 17 years of study, we trapped during 34 trapping sessions and our trapping effort was 143,424 trap nights.

Detection history matrix for capture-mark-recapture (CMR) was prepared traditionally. We considered the capture of an individual by at least one trap in a single night as a detection event for that individual (coded "1"); and, non-detection event if not captured (coded "0"). We collapsed multiple detection events within a secondary occasion into one detection event per individual. The detection-non detection data of each individual per night was pooled across all the locations to record a single event (either detection or non-detection) for that individual. These detection-non detection records for all captured individuals were collated to prepare individual detection history for all species. The final detection history matrix for CMR included unique individuals in rows and sampling occasions (trapping nights) in columns (Williams et al., 2002).

The superpopulation model is a reparameterization of the Jolly-Seber open-population CMR model that estimates i) superpopulation size $(N)$, which is the total number of each species using the study area between 1997 – 2013; ii) apparent survival probability $\left( \phi\right)$, which is the probability of species surviving and returning to the study area between successive sampling occasions; iii) capture probability $(p)$, which is the probability with which a species is captured; and iv) entry probability $(pent)$, which is the probability that a species from the superpopulation entered the study area between two successive sampling seasons (Williams et al. 2002). The time-specific population size, which was our parameter of interest, and the number of births were then estimated as derived parameters (Schwartz & Arnason, 1996; Williams et al., 2002). Since the trapping protocol and sampled area did not change during this study, the population size also reflects the population density.

Model structure varied among species, depending on the sample size. Initial capture probability of all the species was set at $p\left( 1 \right)=1$. First, we fit models where the capture and survival probabilities were allowed to be affected by only single variables such as time (where time refers to sampling occasions; Spring 1997 = occasion 1; Fall 2013 = occasion 34), season, and sex. Then we fit a series of models where the capture and survival probabilities were influenced by additive or interactive effects of season and sex. For entry probability, we only considered models where the parameter was allowed to be influenced by time, for all species. Also, the superpopulation size was modeled as sex-specific for all species. We obtained estimates of all parameters for each species, including the population size, based on the most parsimonious model, which was selected on the basis of Akaike’s Information Criterion corrected for small sample size (Burnham & Anderson, 2002; Williams et al., 2002). Analyses were carried out using program MARK (White & Burnham, 1999) version 6.2 via the RMark package (Laake, 2013) in program R v. 4.2.2 (R Core Team, 2022).

**Results**

The top model for the big-eared woodrat included time as an influential covariate for $\phi$ and the additive and interactive effects of season and sex as an important covariate for $p$. For three species (California mouse, pinyon mouse and brush mouse), time was an influential covariate for $\phi$ and $p$ was influenced primarily by sex for all these species. For pocket mouse, the top model included sex as the most influencing covariate for both $\phi$ and $p$. $N$ and $pent$ for all species depended on sex and time, respectively. Altogether, based on the top models for each species, evidence was strong that $\phi$ varied across time (collective model weight > 0.99 for models with time as a covariate for $\phi$ for all species), except for pocket mouse whose survival was primarily influenced by the sex of the animals (collective model weight = 0.737 for top two models with sex as a covariate). Table S3 provides information on the structure and strength of the top five models for each species.

**Table S3.** Model comparison for POPAN analysis for the big-eared woodrat (NEMA) and other core species, including the pinyon mouse (PETR), brush mouse (PEBO), California mouse (PECA), and pocket mouse (CHCA) during the spring (reproductive, May) and fall (non-reproductive, October) seasons at Camp Roberts, California. We ran models including effects of sex, time (sampling occasion), and season to determine the most parsimonious model. $\phi$ is the survival probability, $p$ is the capture probability, $pent$ is the probability of entrance and $N$ is the superpopulation. A dot (.) indicates constant parameter and an asterisk sign (*) indicates interactive effect of the covariates involved in the model.

| Species | Model | AICc | ΔAICc | Weight | K |
| --- | --- | --- | --- | --- | --- |
| NEMA | $\phi$(time) $p$(season*sex) $pent$(time) $N$(sex) | 30104.010 | 0 | 1 | 72 |
|  | $\phi$(sex) $p$(season*sex) $pent$(time) $N$(sex) | 30137.860 | 33.842 | 4.48E-08 | 41 |
|  | $\phi$(season) $p$(season*sex) $pent$(time) $N$(sex) | 30209.050 | 105.036 | 0 | 41 |
|  | $\phi$(time) $p$(sex) $pent$(time) $N$(sex) | 30320.800 | 216.787 | 0 | 70 |
|  | $\phi$(sex) $p$(season) $pent$(time) $N$(sex) | 30341.270 | 237.253 | 0 | 39 |
| PETR | $\phi$(time) $p$(sex) $pent$(time) $N$(sex) | 6413.462 | 0 | 0.419 | 70 |
|  | $\phi$(time) $p$(season) $pent$(time) $N$(sex) | 6413.529 | 0.067 | 0.405 | 70 |
|  | $\phi$(time) $p$(season*sex) $pent$(time) $N$(sex) | 6415.187 | 1.725 | 0.177 | 72 |
|  | $\phi$(season) $p$(sex) $pent$(time) $N$(sex) | 6433.314 | 19.852 | 2.05E-05 | 39 |
|  | $\phi$(season) $p$(season) $pent$(time) $N$(sex) | 6433.449 | 19.987 | 1.91E-05 | 39 |
| PEBO | $\phi$(time) $p$(sex) $pent$(time) $N$(sex) | 3518.416 | 0 | 0.613 | 70 |
|  | $\phi$(time) $p$(season*sex) $pent$(time) $N$(sex) | 3519.340 | 0.924 | 0.386 | 72 |
|  | $\phi$(time) $p$(season) $pent$(time) $N$(sex) | 3533.938 | 15.522 | 0.0003 | 70 |
|  | $\phi$(season) $p$(sex) $pent$(time) $N$(sex) | 3543.786 | 25.369 | 1.90E-06 | 39 |
|  | $\phi$(season) $p$(season*sex) $pent$(time) $N$(sex) | 3544.831 | 26.415 | 1.13E-06 | 41 |
| PECA | $\phi$(time) $p$(sex) $pent$(time) $N$(sex) | 2143.889 | 0 | 0.503 | 70 |
|  | $\phi$(time) $p$(season) $pent$(time) $N$(sex) | 2144.314 | 0.426 | 0.407 | 70 |
|  | $\phi$(time) $p$(season*sex) $pent$(time) $N$(sex) | 2147.335 | 3.446 | 0.089 | 72 |
|  | $\phi$(season) $p$(sex) $pent$(time) $N$(sex) | 2188.554 | 44.665 | 1.01E-10 | 39 |
|  | $\phi$(season) $p$(season) $pent$(time) $N$(sex) | 2189.009 | 45.120 | 8.02E-11 | 39 |
| CHCA | $\phi$(sex) $p$(sex) $pent$(time) $N$(sex) | 1754.177 | 0 | 0.483 | 39 |
|  | $\phi$(sex) $p$(season*sex) $pent$(time) $N$(sex) | 1755.459 | 1.283 | 0.254 | 41 |
|  | $\phi$(time) $p$(sex) $pent$(time) $N$(sex) | 1756.291 | 2.114 | 0.168 | 70 |
|  | $\phi$(time) $p$(season) $pent$(time) $N$(sex) | 1758.563 | 4.386 | 0.054 | 70 |
|  | $\phi$(season) $p$(season*sex) $pent$(time) $N$(sex) | 1760.041 | 5.865 | 0.026 | 41 |

Estimates of demographic parameters based on the top model for each species over the entire survey duration are presented in Figs. S2 – S4. Estimate of abundance for these species are presented in the main text (Fig. 3).

Figure S2. Apparent urvival $(\phi)$ estimates for the five species of small mammals during each season of each year for the entire study duration in Camp Roberts, California, 1997 - 2013. Species are: big-eared woodrat (NEMA), pinyon mouse (PETR), brush mouse (PEBO), California mouse (PECA) and pocket mouse (CHCA).

Figure S3. Estimates of the number of births $(B)$ for the five species of small mammals during each season of each year for the entire study duration in Camp Roberts, California, 1997 - 2013. Species are big-eared woodrat (NEMA), pinyon mouse (PETR), brush mouse (PEBO), California mouse (PECA) and pocket mouse (CHCA).

Figure S4. Estimates of the capture probability (*p*) for species of small mammals each season for the entire survey duration in Camp Roberts, California, 1997 - 2013. Species are big-eared woodrat (NEMA), pinyon mouse (PETR), brush mouse (PEBO), California mouse (PECA) and pocket mouse (CHCA).

# References

Burnham, K. P., & Anderson, D. R. (2002). Model selection and inference: A practical information-theoretic approach. Springer.

Laake, J. (2013). RMark: An R interface for analysis of capture-recapture data with MARK. AFSC Processed Rep. 2013-01, 25 p. Alaska Fish. Sci. Cent., NOAA, Natl. Mar. Fish. Serv., 7600 Sand Point Way NE, Seattle WA 98115.

R Development Core Team. (2022). R: A Language and Environment for Statistical Computing. R Foundation for Statistical Computing. Vienna, Austria. [http://www.R-project.org/](http://www.r-project.org/).

White, G.C., & Burnham, K.P. (1999). Program mark: Survival estimation from populations of marked animals. Bird Study, 46, S120–S139.

Williams, B.K., Nichols, J.D., & Conroy, M.J. (2002). *Analysis and management of animal*

*populations*. Academic Press.
